# Supplementary material for: Improving rheumatic fever surveillance in New Zealand: results of a surveillance sector review
Source: BMC Public Health. 2014 May 29;14:528. doi: 10.1186/1471-2458-14-528 (PMC4049392; doi:10.1186/1471-2458-14-528)
Supplement: Additional file 1: Table S1 — Specific rheumatic fever surveillance systems identified. [file 1471-2458-14-528-S1.docx]

**Oliver J, Pierse N, Baker MG.**

MS: 1559743671192688

| **ASK THIS OF THE MANUSCRIPT** | **THIS SHOULD BE INCLUDED IN THE MANUSCRIPT** | **CRITERIA MET**  **(Y/N)** | **Page, paragraph where included** |
| --- | --- | --- | --- |
| **R Relevance of study question** |  |  |  |
| Is the research question interesting?  Is the research question relevant to clinical practice, public health, or policy? | Research question explicitly stated  Research question justified and linked to the existing knowledge base (empirical research, theory, policy) | **Y** | Page 5, paragraph 3  Page 5, paragraphs  2 & 3 |
| **A Appropriateness of qualitative method** |  |  |  |
| Is qualitative methodology the best approach for the study aims?   - *Interviews:* experience, perceptions, behaviour, practice, process - *Focus groups:* group dynamics, convenience, non-sensitive topics - *Ethnography:* culture, organizational behaviour, interaction - *Textual analysis:* documents, art, representations, conversations | Study design described and justified i.e., why was a particular method (e.g., interviews) chosen? | **Y** | Page 6, paragraph 2 |
| **T Transparency of procedures**  *Sampling* |  |  |  |
| Are the participants selected the most appropriate to provide access to the type of knowledge sought by the study?  Is the sampling strategy appropriate? | Criteria for selecting the study sample justified and explained   - *theoretical:* based on preconceived or emergent theory - *purposive:* diversity of opinion - *volunteer:* feasibility, hard-to-reach groups | **Y** | Page 6, paragraph 2 |
| *Recruitment* |  |  |  |
| Was recruitment conducted using appropriate methods? | Details of how recruitment was conducted and by whom | **Y** | Page 6, paragraph 2 |
| Is the sampling strategy appropriate?  Could there be selection bias? | Details of who chose not to participate and why | **Y** | Added on Page 7, paragraph 3 |
| *Data collection* |  |  |  |
| Was collection of data systematic and comprehensive? | Method(s) outlined and examples given (e.g., interview questions) | **Y** | Page 7, paragraphs  3 & 4,  Page 8, paragraph 1 |
| Are characteristics of the study group and setting clear? | Study group and setting clearly described | **Y** | Page 6, paragraph 2 |
| Why and when was data collection stopped, and is this reasonable? | End of data collection justified and described | **Y** | Added on Page 7, paragraph 3 |
| *Role of researchers* |  |  |  |
| Is the researcher(s) appropriate? How might they bias (good and bad) the conduct of the study and results? | Do the researchers occupy dual roles (clinician and researcher)? Are the ethics of this discussed? Do the researcher(s) critically examine their own influence on the formulation of the research question, data collection, and interpretation? | **Y** | The interviewer did not occupy dual roles. This research formed the basis of a Ministry of Health report. The study aims were developed in conjunction with the Ministry, who also helped identify key informants and priorities for improvement. Despite this, the research team consulted with each other independently at all stages during this study (Page 6, paragraph 3).  Referred to on Page 8, paragraph 1 |
| *Ethics* |  |  |  |
| Was informed consent sought and granted? | Informed consent process explicitly and clearly detailed | **Y** | Added Page 7, paragraph 3 |
| Were participants’ anonymity and confidentiality ensured? | Anonymity and confidentiality discussed | **Y** | Added Page 7, paragraph 3 |
| Was approval from an appropriate ethics committee received? | Ethics approval cited | **Y** | Page 6, paragraph 3 |
| **S Soundness of interpretive approach**  *Analysis* |  |  |  |
| Is the type of analysis appropriate for the type of study?   - *thematic:* exploratory, descriptive, hypothesis generating - *framework:* e.g., policy - *constant comparison/grounded theory:* theory generating, analytical   Are the interpretations clearly presented and adequately supported by the evidence? | Analytic approach described in depth and justified  *Indicators of quality:* Description of how themes were derived from the data (inductive or deductive)  Evidence of alternative explanations being sought  Analysis and presentation of negative or deviant cases | **Y** | Page 8, paragraphs 4 & 5 |
| Are quotes used and are these appropriate and effective? | Description of the basis on which quotes were chosen  Semi-quantification when appropriate  Illumination of context and/or meaning, richly detailed | **Y** | Justified quotes are widely used: Page 13, paragraphs 1, 2 & 4, page 14, paragraphs 4 & 5, page 15, paragraph 1. |
| Was trustworthiness/reliability of the data and interpretations checked? | Method of reliability check described and justified e.g., was an audit trail, triangulation, or member checking employed? Did an independent analyst review data and contest themes? How were disagreements resolved? | **Y** | Page 8, paragraph 4 |
| *Discussion and presentation* |  |  |  |
| Are findings sufficiently grounded in a theoretical or conceptual framework?  Is adequate account taken of previous knowledge and how the findings add? | Findings presented with reference to existing theoretical and empirical literature, and how they contribute | **Y** | Page 8, paragraphs 4 & 5  Previous knowledge discussed: Page 4 paragraph 5 and page 6, paragraph 1  How findings add: Page 17, paragraph 1 |
| Are the limitations thoughtfully considered? | Strengths and limitations explicitly described and discussed | **Y** | Pages 16 - 17 |
| Is the manuscript well written and accessible? | Evidence of following guidelines (format, word count)  Detail of methods or additional quotes contained in appendix  Written for a health sciences audience | **Y** | Throughout manuscript  Appendix available |
| Are red flags present? These are common features of ill-conceived or poorly executed qualitative studies, are a cause for concern, and must be viewed critically. They might be fatal flaws, or they may result from lack of detail or clarity. | *Grounded theory:* not a simple content analysis but a complex, sociological, theory generating approach  *Jargon:* descriptions that are trite, pat or jargon filled should be viewed sceptically  *Over interpretation:* interpretation must be grounded in "accounts" and semi-quantified if possible or appropriate  *Seems anecdotal, self evident:* may be a superficial analysis, not rooted in conceptual framework or linked to previous knowledge, and lacking depth  *Consent process thinly discussed:* may not have met ethics requirements  *Doctor-researcher:* consider the ethical implications for patients and the bias in data collection and interpretation | **Y** | A complex, semi-quantified thematic analysis is described in the Results Section. |
